# Neck adiposity on standard oncologic CT predicts radiation-induced carotid disease in oropharyngeal cancer

**DOI:** 10.1186/s40959-026-00486-y

**Published:** 2026-04-25

**Authors:** Efthymios Triantafyllou, Dhawal S. Bandrey, Bhumiben Patel, Patrik J. Van-Bergen, Gabrielle N. Mbagwu, Jennifer Shen, Taher K. Kapadia, Juhee Song, Jun-ichi Abe, Amy C. Moreno, Mohamed A. Naser, Katherine A. Hutcheson, David I. Rosenthal, Shaden Khalaf, Cezar Iliescu, Anita Deswal, Elie Mouhayar, Clifton D. Fuller, Efstratios Koutroumpakis

**Affiliations:** 1 Department of Cardiology, Division of Internal Medicine, The University of Texas MD Anderson Cancer Center, 1515 Holcombe Blvd # 1451, Houston, TX 77030 USA; 2 Department of Internal Medicine, The University of Texas at Houston, Houston, TX USA; 3 Department of Biostatistics, The University of Texas MD Anderson Cancer Center, Houston, TX USA; 4 Department of Radiation Oncology, The University of Texas MD Anderson Cancer Center, Houston, TX US; 5 Department of Head and Neck Surgery, Division of Surgery, The University of Texas MD Anderson Cancer Center, Houston, TX USA

**Keywords:** Cerebrovascular events, Perivascular fat, Head and neck cancer, Radiation therapy, Deep learning model, Artificial intelligence

## Abstract

**Background:**

Bidirectional signaling between arteries and adjacent adipose tissue has been implicated in atherosclerosis progression; we sought to ascertain whether baseline neck adiposity could be associated *in vivo with* radiation therapy (RT) induced carotid artery disease (RICAD) and correlated endpoints.

**Objectives:**

To evaluate whether neck adiposity at the time of cancer diagnosis is associated with the subsequent development of RICAD in patients with oropharyngeal cancer (OPC).

**Methods:**

Patients with OPC treated with RT at a tertiary center between 2005 and 2010 were identified. A validated deep learning algorithm was applied to pre-treatment neck CT scans to quantify adipose tissue at the C3 vertebral level.

**Results:**

Of 231 patients, 91% were male and 97% white, with a median age of 57 (IQR 52–63) years at diagnosis. Mean BMI was 28.7 ± 4.86 kg/m². With a median follow-up of 14.3 years (IQR 9.9–16.3), 44% developed new or worsened carotid atherosclerosis, 6% carotid stenosis > 50% and 6.5% stroke/TIA, while 45% developed the composite endpoint. Approximately 39% died. In the multivariable analysis, neck adiposity area index was significantly associated with the composite event (subdistribution hazard ratio (sHR) 1.05 per unit increase, 95% CI 1.02–1.07, *p* < 0.001). Baseline use of antiplatelets and tumor subsite (tonsil vs. other than tonsil) were also associated with the composite endpoint, while skeletal muscle index and traditional vascular risk factors were not.

**Conclusions:**

Pre-treatment neck adiposity area index measured at the C3 vertebral level on standard of care oncologic CT scans predicts RICAD in OPC survivors.

**Supplementary Information:**

The online version contains supplementary material available at 10.1186/s40959-026-00486-y.

## Background

Radiation therapy (RT)-induced carotid artery disease (RICAD) is a serious late complication in patients with oropharyngeal cancer (OPC) - the most common type of head and neck cancer. With 5-year survival rates approaching 80%, the focus has shifted from cancer treatment to the prevention of treatment-related complications [1]. The reported 10-year incidence of asymptomatic carotid stenosis is approximately 30%, and transient ischemic attack (TIA) or stroke 10%, underscoring the significant contribution of RICAD in the morbidity and mortality of this population [2].

Traditional vascular risk factors have been inconsistently linked to RICAD, highlighting the need for further understanding of disease pathophysiology [3]. Although several factors, such as radiotherapy dose, chemotherapy type and age, have been proposed to contribute to the pathogenesis of RICAD their exact role remains unclear. In the past decades, adipose tissue has emerged as a key component of atherosclerosis and cardiovascular morbidity. Multiple studies have shown that there is a bidirectional relationship between adipose tissue and vascular endothelium mediated through vasoactive adipokines and cytokines [4–6]. Coronary CT angiography has been used to risk stratify patients, as it can detect variations in perivascular adipose tissue density, which indicate active inflammation and plaques vulnerability [7–9]. Similar to the coronary arteries, the carotid arteries interact with the surrounding neck adipose tissue, highlighting its potential contributions to the pathogenesis of the disease [10].

Radiation directly damages both endothelial cells and adipose tissue by promoting oxidative stress and senescence [11, 12]. Neck adiposity may act synergistically with radiation and cause chronic inflammation and secretion of adipokines that accelerate atherosclerosis and vascular injury. Whether these findings will translate to the clinical setting is not known. The aim of this study was to evaluate the relationship between increased neck adipose tissue -quantified using baseline pre-RT CT scans -and RICAD.

## Methods

### Patient population

Consecutive patients diagnosed with OPC and treated with RT at a single tertiary cancer center between 2005 and 2010 were retrospectively identified. Baseline demographic and clinical characteristics, including age, sex, tumor site, RT dose, cardiovascular risk factors, history of cardiovascular disease and cerebrovascular events were extracted from electronic medical records. Cardiovascular risk factors were defined as a prior clinical diagnosis with ongoing treatment at baseline, independent of disease control status. OPC staging was based on the 7th edition of American Joint Committee on Cancer (AJCC) staging system using pretreatment imaging scans. Institutional Review Board approved this retrospective study (PA 2025 − 0187) and waived the requirement for informed consent, as the research involved no greater than minimal risk for the subjects.

### Adipose tissue and skeletal muscle quantification

Adipose tissue and skeletal muscle quantification was performed at the C3 vertebral level based on prior reports showing comparable accuracy to the conventional L3 abdominal level in predicting outcomes [13]. The C3 level was chosen due to the frequent absence of abdominal CT imaging in this population, offering a more practical and widely applicable alternative for body composition assessment.

**Fig. 1:**
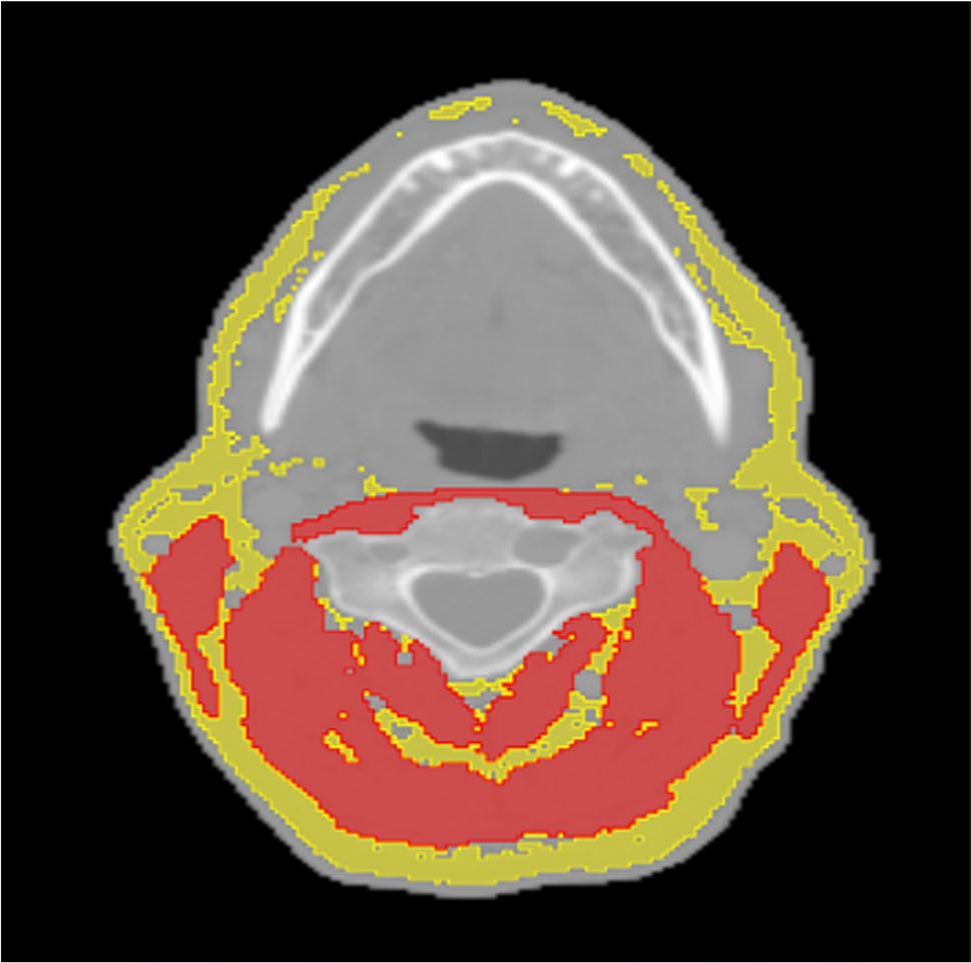
Tissue segmentation using a validated deep learning model. Representative axial CT image at the C3 vertebral level showing segmented adipose tissue (yellow) and skeletal muscle (red)

A previously validated deep learning model for skeletal muscle segmentation at the C3 vertebral level [14] was adapted to automatically segment skeletal muscle and adipose tissue on pre-treatment planning CT scans of OPC patients (Fig. 1). As a first step, a 3D ResUNet model located the C3 vertebral region and selects the mid-axial slice. Then a 2D ResUNet model performed auto-segmentation of skeletal muscle and adipose tissue on this one slice only. The cross-sectional areas of each tissue were computed and normalized to patient height squared, to derive the adipose tissue and skeletal muscle indices. The median adipose tissue density values (Hounsfield Units) for the adiposity area were extracted.

### Endpoints

The primary endpoint was time to development of the composite outcome of: (i) new or worsened carotid atherosclerosis, (ii) carotid stenosis greater than 50%, (iii) TIA or stroke. Clinically indicated CT scans were identified and used to assess atherosclerosis progression across the entire cohort. The timing of CT scans varied across patients based on clinical need. For each patient, the most recent available CT scan was compared to the baseline pretreatment scan to determine atherosclerosis progression. A single investigator reviewed all baseline and follow-up standard-of-care oncologic CT scans and defined atherosclerosis progression as worsening of carotid calcifications on follow-up CT imaging compared to baseline. Additionally, worsening findings on carotid ultrasound, based on the available reports, were also included in this definition. Carotid stenosis greater than 50% was defined using standard definitions [15]. Stroke was defined as imaging (CT or MRI) confirmed infarction, while TIA was characterized by transient neurological symptoms (weakness, hemiplegia, paralysis, aphasia, amaurosis fugax) documented in the medical record and labeled as TIA.

Secondary endpoints comprised time to development of each individual component of the composite outcome: (i) new or worsening carotid atherosclerosis, (ii) carotid stenosis > 50%, (iii) stroke/TIA. All-cause mortality was also assessed as a secondary endpoint.

### Statistical analysis

Categorical variables are described as frequencies (percentages), while continuous variables are summarized as mean ± standard deviation or median (interquartile range [IQR], for non-normally distributed variables). Time to event was calculated from the start of RT to the occurrence of the first event of interest. Event of interest includes development of the composite outcome of new or worsened carotid atherosclerosis, stenosis greater than 50%, TIA or stroke. Patients without an event were censored at the time of their last follow-up or death, with death treated as competing-risk event. Fine-Gray models were used to identify factors associated with the cumulative incidence of the primary and secondary endpoints. The variables that came out significant in the univariate model were considered in the multivariable analysis. Multivariable Fine-Gray models were constructed to evaluate the association of baseline demographic and clinical variables with the endpoints. When examining the association between BMI or adiposity area index with the endpoints, given their collinearity, separate multivariable Fine-Gray models were constructed. To evaluate whether there is additive value from adiposity area index, a more specific but more laborious index to calculate, over the widely used BMI in their association with radiation-induced cerebrovascular events, the Bayesian Information Criterion (BIC) was calculated to compare model performance, with the lowest BIC value indicating the best overall fit for the primary endpoint.

The cumulative incidence of the composite event was estimated using the Aalen-Johansen method and the survival probability was estimated by Kaplan Meier method. Comparisons between patients with and without radiation-induced vascular adverse events were conducted using two-sample t-tests or Wilcoxon rank-sum tests for continuous variables, and Chi-square tests or Fisher’s exact tests for categorical variables. A p-value of less than 0.05 was considered statistically significant. All analyses were performed using SAS 9.4 (SAS Institute INC, Cary, NC).

## Results

### Cohort characteristics

A total of 231 patients with OPC treated with RT at a tertiary cancer center between 2005 and 2010 were identified. The majority were male (90.9%) and white (97.4%) with a median age of 57 years (IQR 52–63) at cancer diagnosis. The mean body mass index (BMI) was 28.7 ± 4.86 kg/m². Prior to RT, 42% had hypertension, 27.7% hyperlipidemia, 11.7% diabetes and 53.2% were active or former smokers. Preexisting cardiovascular disease was present in 18.2% of patients, with coronary artery disease accounting for approximately 8% of these cases. Statins were used by 26.8% of patients and antiplatelet therapy by 16%. 64% of the cohort had T1 or T2 tumors and 92% lymph node metastasis (N1, N2 and N3). HPV status was available for 103 patients, of whom 89 were HPV-positive and 14 were HPV-negative. All patients were treated with Intensity-Modulated Radiation Therapy (IMRT), either alone or in combination with chemotherapy, while none underwent oncologic surgery. The mean RT dose was 68.8+/-2.51 Gy. Overall, 47.2% received concurrent chemoradiotherapy, 28.1% received induction chemotherapy followed by concurrent chemoradiotherapy, 12.1% received induction chemotherapy followed by radiation and 12.6% received radiation alone. Additional demographic and clinical parameters are summarized in Table 1.

**Table 1:** Patient baseline characteristics and cerebrovascular outcomes (page 9)

Covariate (*N* = 231)	*N* (%), median (IQR) or mean (SD)
Age at RT (in years), median (IQR)	57 (52–63)
Female sex	21 (9.1)
White race	225 (97.4)
Hispanic	5 (2.2)
BMI (kg/m²), mean (SD)	28.7 (4.86)
History of CV risk factors	206 (89.2)
HTN	97 (42.0)
DLD	64 (27.7)
DM	27 (11.7)
Smoking	123 (53.2)
History of CVD	42 (18.2)
CAD	19 (8.2)
Statin use	62 (26.8)
Antiplatelet use	37 (16.0)
Tumor Laterality	
L	120 (51.9)
Midline	5 (2.2)
NOS	1 (0.4)
R	105 (45.5)
Tumor subsite	
Tonsil	90 (40.7)
Other	131 (59.3)
T stage	
T1	41 (17.7)
T2	106 (45.9)
T3	54 (23.4)
T4	30 (13.0)
N stage	
N0	19 (8.2)
N1	20 (8.7)
N2	187 (81.0)
N3	5 (2.2)
Treatment scheme	
Concurrent chemoradiotherapy	109 (47.2)
Induction chemotherapy + concurrent chemoradiotherapy	65 (28.1)
Induction chemotherapy+Radiation alone	28 (12.1)
Radiation alone	29 (12.6)
RT dose (Gy), mean (SD)	68.8 (2.51)
RT number of fractions	32.9 (2.86)
Progression of carotid atherosclerosis	102 (44.2)
New carotid stenosis > 50%	14 (6.1)
New TIA/Stroke	15 (6.5)

*Abbreviations*: *RT *Radiotherapy, *BMI *Body Mass Index, *CV *Cardiovascular, *HTN *Hypertension, *DLD *Dyslipidemia, *DM *Diabetes, *CVD *Cardiovascular Disease, *CAD *Coronary Artery Disease, *NOS *Not otherwise specified, *TIA *Transient Ischemic Attack

The mean adiposity area at C3 vertebral level was 39.1 (29.7–55.6) cm², adiposity area index was 12.5 (9.6–18.6) cm²/m², SM area at C3 was 46.4 (40.2–50.9) cm² and the SM index 14.7 (13.2–16.5) cm²/m², as calculated in the baseline CT scan before the initiation of RT (Table 2).

**Table 2:** Adiposity and skeletal muscle measurements (page 9)

Covariate	Median (IQR)
Adiposity density (HU)	-78 (-84 - -71)
Adiposity area at C3 (cm²)	39.1 (29.7–55.6)
Adiposity area index (cm²/m²)	12.5 (9.6–18.6)
SM area at C3 (cm²)	46.4 (40.2–50.9)
SM index (cm²/m²)	14.7 (13.2–16.5)

*Abbreviations*: *SM *Skeletal muscle, *HU *Hounsfield Units

### Cerebrovascular events and mortality

**Fig. 2:**
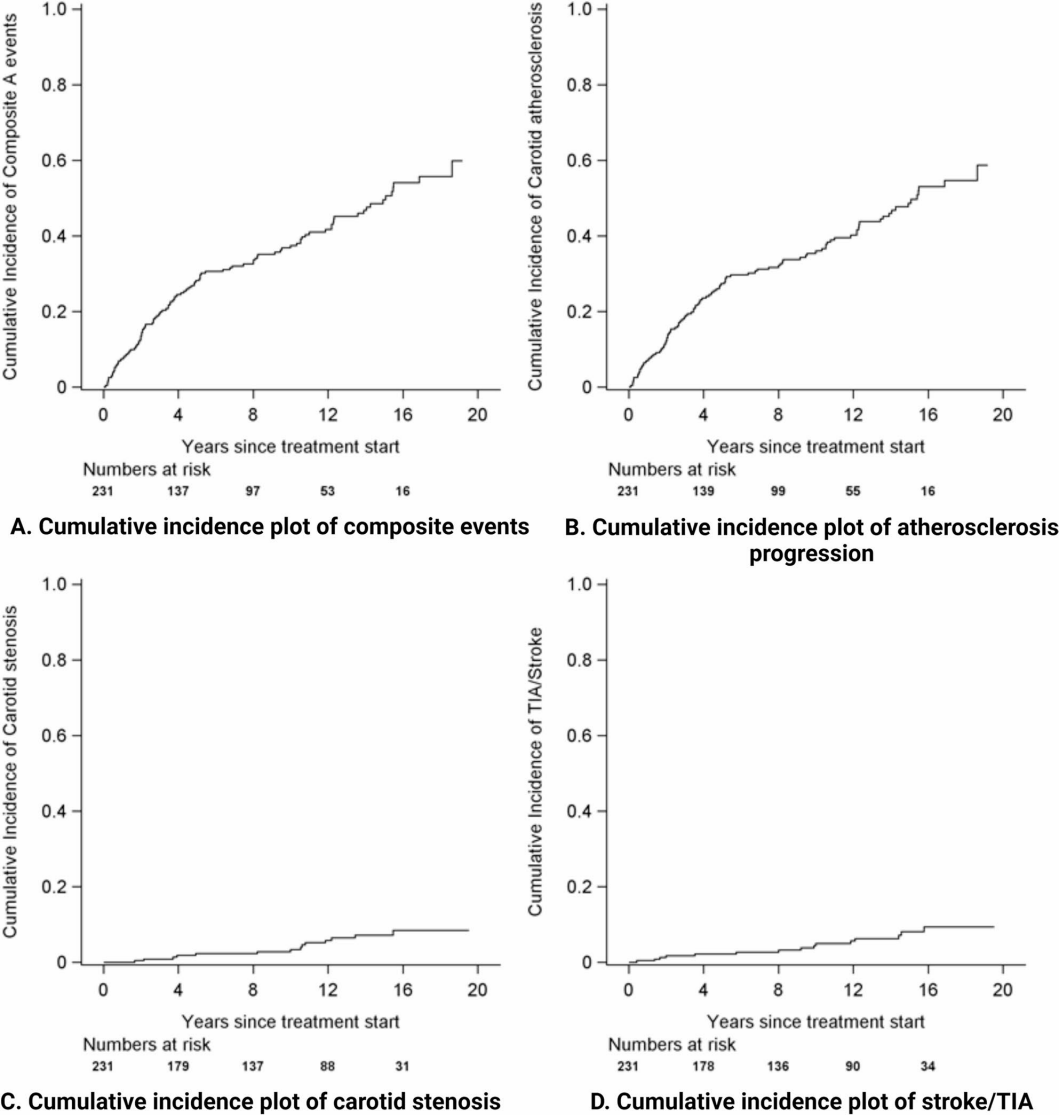
Incidence plots of the primary and secondary endpoints. Plot **A** reflects the cumulative incidence of the composite endpoint, plot **B** the incidence of progression of atherosclerosis, plot **C** the incidence of carotid stenosis > 50% and plot **D** the incidence of stroke/TIA

During a median follow-up of 14.3 years (IQR 9.9–16.3), the composite endpoint of new TIA/stroke, progression of carotid atherosclerosis and carotid stenosis more than 50%, occurred in 45% of patients (Fig. 2). A total of 102 patients (44.2%) demonstrated progression of carotid atherosclerosis and 14 (6.1%) developed new carotid stenosis greater than 50% at a median time of 3 years (IQR 1-8.25) from RT, based on the most available CT or ultrasound. Fifteen patients (6.5%) experienced new stroke or TIA at a median time of 9 years (IQR 3.5-11.75) from RT (Table 1). Overall, 91 patients (39.4%) died during follow-up.

### Clinical predictors of primary and secondary endpoints

In the multivariable Fine Gray analysis, accounting for death as a competing risk, higher adiposity area index (sHR = 1.05, 95%CI 1.02–1.07, *p* < 0.001), use of antiplatelets (sHR = 1.88, 95% CI 1.20–2.96, *p* = 0.006) and tumor subsites other than tonsil, including the tongue, palate and posterior pharyngeal wall, (sHR = 1.51, 95%CI 1.01–2.25, *p* = 0.046) were independently associated with the cumulative incidence of primary composite endpoint (Table 3). In contrast, RT dose, smoking status and traditional vascular risk factors were not significantly associated with the cumulative incidence of endpoint. The Bayesian Information Criterion (BIC) was equal to 1004.641 for the multivariable Fine-Grey model using adiposity area index as a predictor (Table 3). For the multivariable model using BMI instead of adiposity area index, BIC was equal to 1006.735 (Supplemental Table 4).

**Table 3:** Multivariable Fine-Gray model for the primary endpoint including Adiposity area index as an exploratory factor. (BIC = 1004.641), (page 10)

	Subdistribution Hazard Ratio (95 CI)	*P*-value^1^
Adiposity area index (cm²/m²) (in 1 unit change)	1.05 (1.02–1.07)	< 0.001
Antiplatelet use	1.88 (1.20–2.96)	0.006
Subsite		
Tonsil	Reference	
Other	1.51 (1.01–2.25)	0.046

^1^Covariate Wald *p*-value

Regarding the secondary endpoints, neither adiposity nor SM index were associated with the development of new carotid stenosis greater than 50% or new TIA/stroke, when studied as independent outcomes. In the multivariate model using progression of atherosclerosis as the endpoint, only adiposity area index (HR 1.06, 95% CI 1.03–1.08, *p* < 0.001), non-white race (HR 6.47, 95% CI 2.31–18.10, *p* < 0.001) and tumor subsites other than the tonsil (HR 1.57, 95% CI 1.04–2.38, *p* = 0.03) were significant.

In a sensitivity analysis comparing patients who developed the composite event with those who did not, significant differences were noted in adiposity area at C3 (median: 42.8 vs. 35.2 cm², *p* = 0.001) and adiposity area index (median: 14.0 vs. 11.6 cm²/m², *p* = 0.002). Additional differences were observed in BMI (mean: 29.8 vs. 27.8 kg/m², *p* = 0.003), prevalence of coronary artery disease (12.5% vs. 4.7%, *p* = 0.032) and antiplatelet use (24% vs. 9.4%, *p* = 0.003). Traditional cardiovascular risk factors, prior history of cardiovascular disease, SM area at C3 vertebral level and SM index did not differ significantly between groups. 

## Discussion

To our knowledge, this is the first study to evaluate the role of neck adipose tissue -quantified using a deep learning algorithm applied to the standard of care pre-treatment oncologic CT scans -in the development of RICAD among patients with OPC treated with RT. In this retrospective study, we found that: (1) A higher adiposity area index at the C3 vertebral level was associated with the composite of progression of carotid atherosclerosis, carotid stenosis > 50% and stroke/TIA, (2) Adiposity area index performed better than BMI in predicting the composite endpoint, and (3) Traditional vascular risk factors, such as hypertension, dyslipidemia, diabetes and smoking were not associated with the composite outcome in our analysis.

In a general population study, Baradaran et al. showed that patients with carotid stenosis and ipsilateral stroke had higher pericarotid adipose tissue density than asymptomatic patients. Furthermore, subjects with carotid stenosis had higher perivascular adipose tissue density compared to those with non-stenotic carotid arteries [10]. Despite this study in the general population, there are no reports to our knowledge examining the association of neck adiposity with RICAD and cerebrovascular events. In our cohort, greater neck adiposity area index was associated with the composite outcome, driven primarily by progression of carotid atherosclerosis. This finding supports the hypothesis that adipose tissue may serve as a substrate for atherosclerosis progression following RT [4]. The adiposity area index was not significantly associated with the other components of the composite outcome—development of > 50% carotid stenosis or stroke/TIA— likely due to the relatively small sample size and low event rates. Nonetheless, atherosclerosis progression is a precursor to clinically significant carotid stenosis and, ultimately TIA or stroke [16]. Of note, our observed rates of carotid stenosis and TIA/stroke over 10 years were lower than those reported in prior studies [2, 17, 18]. This likely reflects the fact that many patients seek cancer treatment at our tertiary center but return to local providers for survivorship care, resulting in incomplete ascertainment of long-term events. In addition, the absence of evidence-based surveillance guidelines for carotid ultrasonography after RT has resulted in inconsistent imaging practices, which likely contributed to the lower observed rate of carotid stenosis in our cohort compared with the literature. Despite these limitations, we were still able to demonstrate a statistically significant association between neck adiposity area index and progression of carotid atherosclerosis. It is also worth noting that, unlike Baradaran et al., who quantified adipose tissue density in a localized region around the carotid artery, our approach measured adipose tissue area and density across the entire cross-sectional area of the neck at the C3 vertebral level. While adiposity area index was significantly associated with the composite outcome, adipose tissue density did not reach statistical significance, which may reflect differences in the spatial specificity of the measurement approach.

Another important observation is that, as adiposity area index and BMI are highly correlated, separate multivariable models were constructed for each variable and the BIC was calculated to compare their performance. Following Raftery’s guideline, a BIC difference between 2 and 5 provides positive evidence favoring the model with the lower BIC; therefore, these results indicate that the model including adiposity area index performs modestly better than the model including BMI for predicting the composite outcome [19]. The findings align with the literature from the general population, where neck circumference serves as a surrogate for adiposity, and support that in multivariable models, neck adipose tissue is independently associated with cerebrovascular risk and performs better than BMI in predicting incident cerebrovascular events [20, 21]. BMI does not provide an accurate assessment of body composition and can often result in misleading risk assessment. Increased adiposity and sarcopenia - often referred to as sarcopenic obesity - can be masked by normal BMI values, particularly in patients with cancer who experience significant muscle loss due to the disease and its various treatments [22]. Consequently, imaging-based body composition can be used to more accurately identify significant associations with clinical outcomes. Additionally, our approach to quantifying neck adipose tissue relied on standard-of-care pre-radiation CT scans, eliminating the need for additional testing and reducing both physical and financial burden. This exemplifies how existing imaging can be leveraged to extract additional clinically relevant information, improving risk assessment and enhancing our understanding of the pathophysiology of treatment-related complications.

Among other clinical predictors, tumor subsites other than tonsil (including the tongue, palate and posterior pharyngeal wall) were significantly associated with the composite endpoint when compared to tonsillar cancer. Even though this finding is unexpected, it is interesting and warrants further investigation in future studies. Furthermore, antiplatelet use was significantly associated with the composite endpoint and remained significant even after adjustment for pre-existing cardiovascular disease. This finding possibly reflects residual confounding from unmeasured variables, undocumented high-risk characteristics, or underlying coronary artery disease, rather than a direct harmful effect of antiplatelet agents.

In this cohort, traditional cardiovascular risk factors - including hypertension, dyslipidemia, diabetes, and smoking - were not associated with the primary endpoint. This observation may suggest that mechanisms distinct from those driving atherosclerosis in the general population may underlie the development of RICAD [11]. Chronic inflammation and fibrosis mediated by perivascular adiposity are likely contributors to RICAD pathogenesis [23]. These findings have important clinical implications for risk stratification and management of OPC survivors following RT.

In prior studies, artificial intelligence-based deep learning algorithms have also been applied to automatically quantify skeletal muscle at the C3 vertebral level in patients with OPC before RT [14]. Most of these studies used CT imaging, although some employed MRI, which offers comparable sensitivity for soft tissue quantification [24]. Sarcopenia, defined as decreased skeletal muscle area and expressed using the skeletal muscle index, has been shown to predict outcomes and overall survival in head and neck cancer patients [25]. In our study, baseline skeletal muscle index was not significantly associated with the composite cerebrovascular endpoint, potentially suggesting that the pathogenetic mechanisms driving sarcopenia in OPC survivors differ from those underlying RICAD. It should be noted that we assessed skeletal muscle mass at baseline only and did not evaluate changes in skeletal muscle index following treatment, which may limit conclusions regarding the relationship between sarcopenia and RICAD in this population.

### Limitations

Our study is subject to limitations. First, it is a retrospective study conducted at a tertiary cancer center, which is inherently prone to selection biases. As mentioned earlier, patients may have received care for non-cancer related conditions at outside hospitals, potentially leading to underreporting of events and inconsistent performance of carotid duplex, which likely explains the low incidence of carotid stenosis. Atherosclerosis progression was determined from clinically indicated oncologic CT scans rather than a standardized surveillance protocol. This approach may introduce ascertainment bias, as patients with more severe illness or clinical concerns may have undergone more frequent imaging, potentially leading to preferential detection of atherosclerotic changes in this subgroup. Furthermore, we quantified neck adiposity at the C3 level but that might not be representative of perivascular adiposity throughout the length of the carotid artery. Additionally, the deep learning model was applied only to baseline pre-therapy CT scans, while body composition changes during or after therapy may influence the progression of atherosclerosis. Larger, multicenter prospective studies with imaging at multiple timepoints are warranted to validate these findings and to determine the role of neck adiposity in RICAD development. Despite its limitations, this is the first study to demonstrate an association between neck adiposity - quantified using deep learning models on standard-of-care CT scans -and subsequent RICAD in patients with oropharyngeal cancer, providing valuable insight into this serious complication of neck irradiation.

## Conclusion

In conclusion, this retrospective study demonstrates that neck adiposity - quantified using deep learning models on standard-of-care CT scans - predicts carotid atherosclerosis progression in OPC survivors treated with radiation therapy, outperforming BMI in predicting the study endpoint. These findings highlight the potential pathogenetic role of neck and perivascular adipose tissue in the development of RICAD and support the use of this measure for risk stratification, surveillance, and management strategies. Moreover, our study underscores the value of artificial intelligence–guided body composition analysis using routine imaging as a practical and scalable tool for identifying high-risk patients.

## Supplementary Information

Supplementary Material 1: Table S1. Univariable Fine-Gray models for the primary endpoint: progression of atherosclerosis, carotid stenosis, stroke and TIA.

Supplementary Material 2: Table S2. STROBE Statement—checklist of items that should be included in reports of observational studies.

Supplementary Material 3: Table S3. Univariate Fine-Gray models for the secondary endpoints.

Supplementary Material 4: Table S4. Multivariable Fine-Gray model for the primary endpoint including BMI as an exploratory factor.

## Data Availability

In accordance with NOT-OD-21-013, Final NIH Policy for Data Management and Sharing, anonymized/de-identified data that support the findings of this study are openly available in an NIH-supported generalist scientific data repository (figshare) at http://doi.org/10.6084/m9.figshare.30826145 no later than the time of an associated publication.

## Abbreviations

BIC: Bayesian Information Criterion
BMI: Body mass index
IMRT: Intensity-Modulated Radiation Therapy
IQR: Interquartile range
OPC: Oropharyngeal cancer
RICAD: Radiation-induced carotid artery disease
RT: Radiation therapy
SM: Skeletal muscle
TIA: Transient ischemic attack
