# Supplementary material for: Neck adiposity on standard oncologic CT predicts radiation-induced carotid disease in oropharyngeal cancer
Source: Cardiooncology. 2026 Apr 25;12:76. doi: 10.1186/s40959-026-00486-y (PMC13248235; doi:10.1186/s40959-026-00486-y)
Supplement: Supplementary file 1 — Supplementary Material 1: Table S1. Univariable Fine-Gray models for the primary endpoint: progression of atherosclerosis, carotid stenosis, stroke and TIA. [file 40959_2026_486_MOESM1_ESM.docx]

| **Supplemental Table 1. Univariable Fine-Gray models for the primary endpoint: progression of atherosclerosis, carotid stenosis, stroke and TIA** | | |
| --- | --- | --- |
|  | Subdistribution Hazard Ratio (95 CI) | P-value |
| Age at RT (in years) (in 1 unit change) | 1.00 (0.98-1.02) | 0.99 |
| Female sex | 0.56 (0.25-1.27) | 0.17 |
| Non-White Race | 1.42 (0.34-5.91) | 0.63 |
| Hispanic | 2.14 (0.60-7.62) | 0.24 |
| BMI (in 1 unit change) | 1.06 (1.01-1.11) | 0.009 |
| History of CV risk factors | 1.26 (0.71-2.27) | 0.43 |
| HTN | 1.06 (0.72-1.55) | 0.78 |
| DLD | 1.00 (0.65-1.53) | 0.99 |
| DM | 1.13 (0.61-2.08) | 0.70 |
| Smoking | 0.96 (0.66-1.41) | 0.85 |
| History of cardiovascular disease | 1.64 (1.02-2.63) | 0.039 |
| CAD | 2.11 (1.13-3.95) | 0.019 |
| Statin use | 1.56 (1.03-2.36) | 0.035 |
| Antiplatelet use | 2.25 (1.43-3.53) | <0.001 |
| Tumor laterality |  |  |
| L | Reference |  |
| Midline | 0.49 (0.07-3.32) | 0.47 |
| R | 1.04 (0.71-1.52) | 0.85 |
| Subsite |  |  |
| Tonsil | Reference |  |
| Other | 1.48 (0.99-2.20) | 0.056 |
| T stage |  |  |
| 1 | Reference |  |
| 2 | 0.77 (0.47-1.29) | 0.32 |
| 3 | 1.23 (0.69-2.18) | 0.48 |
| 4 | 0.86 (0.40-1.82) | 0.69 |
| N stage |  |  |
| 0 | Reference |  |
| 1 | 0.41 (0.13-1.25) | 0.12 |
| 2-3 | 0.66 (0.35-1.25) | 0.20 |
| Therapy |  |  |
| Radiation alone | Reference |  |
| Concurrent chemoradiotherapy | 1.15 (0.60-2.20) | 0.68 |
| Induction chemotherapy + concurrent chemoradiotherapy | 1.17 (0.59-2.30) | 0.65 |
| Induction chemotherapy+Radiation alone | 0.97 (0.43-2.18) | 0.93 |
| RT dose (in 1 unit change) | 0.99 (0.91-1.06) | 0.70 |
| RT number of fractions (in 1 unit change) | 0.96 (0.91-1.02) | 0.23 |
| Adiposity density (HU) (1 unit change) | 0.98 (0.96-1.00) | 0.027 |
| Adiposity area at C3 (cm²) (in 1 unit change) | 1.02 (1.01-1.03) | <0.001 |
| Adiposity area index (cm²/m²) (in 1 unit change) | 1.05 (1.02-1.08) | <0.001 |
| SM area at C3 (cm²) (in 1 unit change) | 1.02 (0.99-1.04) | 0.17 |
| SM index (cm²/m²) (in 1 unit change) | 1.05 (0.99-1.12) | 0.09 |
|  | | |

Abbreviations: BMI-Body Mass Index, CAD-Coronary Artery Disease, CV-Cardiovascular, CVD-Cardiovascular Disease, DLD-Dyslipidemia, DM-Diabetes Melitus, HTN-Hypertension, HU- Hounsfield units, NOS--Not otherwise specified, RT-Radiotherapy
