# Supplementary material for: Neck adiposity on standard oncologic CT predicts radiation-induced carotid disease in oropharyngeal cancer
Source: Cardiooncology. 2026 Apr 25;12:76. doi: 10.1186/s40959-026-00486-y (PMC13248235; doi:10.1186/s40959-026-00486-y)
Supplement: Supplementary file 3 — Supplementary Material 3: Table S3. Univariate Fine-Gray models for the secondary endpoints. [file 40959_2026_486_MOESM3_ESM.docx]

| **Supplemental Table 3A. Univariable Fine-Gray models for progression of atherosclerosis** | | |
| --- | --- | --- |
|  | Subdistribution Hazard Ratio (95 CI) | P-value |
| Age at RT (in years) (in 1 unit change) | 1.00 (0.98-1.02) | 0.8620 |
| Female sex | 0.58 (0.26-1.32) | 0.1976 |
| Non-White Race | 1.47 (0.35-6.20) | 0.5967 |
| Hispanic | 2.29 (0.62-8.38) | 0.2121 |
| BMI (in 1 unit change) | 1.07 (1.02-1.11) | 0.0039 |
| History of CV risk factors | 1.25 (0.71-2.22) | 0.4436 |
| HTN | 1.06 (0.72-1.56) | 0.7764 |
| DLD | 1.05 (0.68-1.62) | 0.8293 |
| DM | 1.18 (0.63-2.18) | 0.6059 |
| Smoking | 0.98 (0.67-1.43) | 0.9016 |
| History of cardiovascular disease | 1.56 (0.96-2.53) | 0.0699 |
| CAD | 2.21 (1.18-4.15) | 0.0138 |
| Statin use | 1.64 (1.08-2.49) | 0.0198 |
| Antiplatelet use | 2.12 (1.33-3.37) | 0.0015 |
| Tumor laterality |  |  |
| L | Reference |  |
| Midline | 0.53 (0.08-3.58) | 0.5127 |
| R | 1.11 (0.75-1.63) | 0.6091 |
| Subsite |  |  |
| Tonsil | Reference |  |
| Other | 4.22 (1.92-9.26) | 0.0003 |
| T stage |  |  |
| 1 | Reference |  |
| 2 | 0.73 (0.44-1.21) | 0.2201 |
| 3 | 1.25 (0.70-2.21) | 0.4541 |
| 4 | 0.86 (0.41-1.82) | 0.6926 |
| N stage |  |  |
| 0 | Reference |  |
| 1 | 0.50 (0.16-1.54) | 0.2257 |
| 2-3 | 0.78 (0.40-1.52) | 0.4693 |
| Therapy |  |  |
| Radiation alone | Reference |  |
| Concurrent chemoradiotherapy | 1.33 (0.68-2.59) | 0.4006 |
| Induction chemotherapy + concurrent chemoradiotherapy | 1.35 (0.68-2.69) | 0.3983 |
| Induction chemotherapy+Radiation alone | 0.97 (0.42-2.23) | 0.9353 |
| RT dose (in 1 unit change) | 0.99 (0.91-1.07) | 0.7222 |
| RT number of fractions (in 1 unit change) | 0.97 (0.92-1.03) | 0.3940 |
| Adiposity area at C3 (cm²) (in 1 unit change) | 1.02 (1.01-1.03) | <.0001 |
| Adiposity area index (cm²/m²) (in 1 unit change) | 1.06 (1.03-1.08) | <.0001 |
| SM area at C3 (cm²) (in 1 unit change) | 1.02 (1.00-1.04) | 0.1090 |
| SM index (cm²/m²) (in 1 unit change) | 1.07 (1.00-1.14) | 0.0408 |
|  | | |

| **Supplemental Table 3B. Univariable Fine-Gray models for new carotid stenosis** | | |
| --- | --- | --- |
|  | Subdistribution Hazard Ratio (95 CI) | P-value |
| Age at RT (in years) (in 1 unit change) | 1.01 (0.94-1.07) | 0.86 |
| Female sex | 0.34 (0.00-2.50) | 0.46 |
| Non-White Race | 1.11 (0.01-8.26) | 0.95 |
| Hispanic | 2.80 (0.37-21.01) | 0.32 |
| BMI (in 1 unit change) | 1.06 (0.98-1.14) | 0.18 |
| History of CV risk factors | 0.44 (0.13-1.49) | 0.19 |
| HTN | 1.26 (0.45-3.56) | 0.66 |
| DLD | 1.44 (0.48-4.32) | 0.51 |
| DM | 3.40 (1.07-10.74) | 0.037 |
| Smoking | 1.11 (0.38-3.23) | 0.85 |
| History of cardiovascular disease | 1.78 (0.57-5.59) | 0.32 |
| CAD | 0.83 (0.11-6.49) | 0.86 |
| Statin use | 1.57 (0.53-4.68) | 0.42 |
| Antiplatelet use | 0.88 (0.20-3.93) | 0.87 |
| Tumor laterality |  |  |
| L | Reference |  |
| Midline | 7.56 (0.82-70.07) | 0.074 |
| R | 1.37 (0.47-4.02) | 0.57 |
| Subsite |  |  |
| Tonsil | Reference |  |
| Other | 0.94 (0.33-2.68) | 0.91 |
| T stage |  |  |
| 1 | Reference |  |
| 2 | 0.88 (0.27-3.57) | 0.85 |
| 3 | 0.82 (0.17-3.86) | 0.80 |
| 4 | 0.18 (0.00-1.85) | 0.29 |
| N stage |  |  |
| 0 | Reference |  |
| 1 | 0.72 (0.07-7.78) | 0.78 |
| 2-3 | 0.53 (0.12-2.33) | 0.41 |
| Therapy |  |  |
| Radiation alone | Reference |  |
| Concurrent chemoradiotherapy | 0.39 (0.09-1.65) | 0.20 |
| Induction chemotherapy + concurrent chemoradiotherapy | 0.60 (0.14-2.62) | 0.50 |
| Induction chemotherapy+Radiation alone | 0.99 (0.21-4.63) | 0.99 |
| RT dose (in 1 unit change) | 0.93 (0.70-1.23) | 0.59 |
| RT number of fractions (in 1 unit change) | 1.01 (0.83-1.24) | 0.90 |
| Adiposity area at C3 (cm²) (in 1 unit change) | 1.00 (0.99-1.02) | 0.80 |
| Adiposity area index (cm²/m²) (in 1 unit change) | 1.02 (0.96-1.08) | 0.57 |
| SM area at C3 (cm²) (in 1 unit change) | 1.01 (0.96-1.06) | 0.69 |
| SM index (cm²/m²) (in 1 unit change) | 1.07 (0.91-1.27) | 0.43 |
|  | | |

| **Supplemental Table 3C. Univariable Fine-Gray models for stroke or TIA** | | |
| --- | --- | --- |
|  | Subdistribution Hazard Ratio (95 CI) | P-value |
| Age at RT (in years) (in 1 unit change) | 0.97 (0.92-1.02) | 0.27 |
| Female sex | 0.32 (0.00-2.83) | 0.44 |
| Non-White Race | 2.62 (0.32-21.21) | 0.37 |
| Hispanic | 8.31 (1.74-39.56) | 0.008 |
| BMI (in 1 unit change) | 1.02 (0.93-1.13) | 0.66 |
| History of CV risk factors | 0.46 (0.13-1.61) | 0.22 |
| HTN | 1.11 (0.40-3.05) | 0.84 |
| DLD | 1.22 (0.42-3.48) | 0.72 |
| DM | 2.03 (0.59-7.04) | 0.26 |
| Smoking | 0.72 (0.26-1.98) | 0.53 |
| History of cardiovascular disease | 1.70 (0.54-5.38) | 0.37 |
| CAD | 1.78 (0.39-8.17) | 0.46 |
| Statin use | 1.82 (0.66-5.04) | 0.25 |
| Antiplatelet use | 2.97 (1.01-8.76) | 0.048 |
| Tumor laterality |  |  |
| L | Reference |  |
| Midline | 1.49 (0.01-11.60) | 0.79 |
| R | 0.60 (0.20-1.62) | 0.35 |
| Subsite |  |  |
| Tonsil | Reference |  |
| Other | 1.06 (0.38-2.95) | 0.91 |
| T stage |  |  |
| 1 | Reference |  |
| 2 | 0.83 (0.22-3.18) | 0.78 |
| 3 | 0.50 (0.09-2.91) | 0.44 |
| 4 | 1.34 (0.27-6.78) | 0.72 |
| N stage |  |  |
| 0 | Reference |  |
| 1 | 0.13 (0.00-1.22) | 0.19 |
| 2-3 | 0.23 (0.08-0.77) | 0.012 |
| Therapy |  |  |
| Radiation alone | Reference |  |
| Concurrent chemoradiotherapy | 0.11 (0.02-0.54) | 0.007 |
| Induction chemotherapy + concurrent chemoradiotherapy | 0.34 (0.09-1.25) | 0.11 |
| Induction chemotherapy+Radiation alone | 0.79 (0.22-2.88) | 0.72 |
| RT dose (in 1 unit change) | 0.92 (0.79-1.05) | 0.22 |
| RT number of fractions (in 1 unit change) | 0.84 (0.63-1.11) | 0.22 |
| Adiposity area at C3 (cm²) (in 1 unit change) | 1.01 (0.98-1.03) | 0.59 |
| Adiposity area index (cm²/m²) (in 1 unit change) | 1.02 (0.95-1.09) | 0.60 |
| SM area at C3 (cm²) (in 1 unit change) | 1.04 (0.99-1.10) | 0.12 |
| SM index (cm²/m²) (in 1 unit change) | 1.13 (0.97-1.32) | 0.12 |
|  | | |
