# Supplementary material for: Neck adiposity on standard oncologic CT predicts radiation-induced carotid disease in oropharyngeal cancer
Source: Cardiooncology. 2026 Apr 25;12:76. doi: 10.1186/s40959-026-00486-y (PMC13248235; doi:10.1186/s40959-026-00486-y)
Supplement: Supplementary file 4 — Supplementary Material 4: Table S4. Multivariable Fine-Gray model for the primary endpoint including BMI as an exploratory factor. [file 40959_2026_486_MOESM4_ESM.docx]

| **Supplemental Table 4. Multivariable Fine-Gray model for the primary endpoint including BMI as an exploratory factor** (BIC=1006.735), (page 10) | | |
| --- | --- | --- |
|  | Subdistribution Hazard Ratio (95 CI) | P-value^1^ |
| BMI (kg/m²) (in 1 unit change) | 1.06 (1.02-1.10) | 0.008 |
| Antiplatelet use | 1.95 (1.25-3.07) | 0.004 |
| Subsite |  |  |
| Tonsil | Reference |  |
| Other | 1.48 (0.99-2.22) | 0.055 |
| ^1^Covariate Wald p-value; | | |

Abbreviations: BMI-Body Mass Index
